# Human Granulocytic Ehrlichiosis Complicating Early Pregnancy

**DOI:** 10.1155/2008/359172

**Published:** 2008-05-22

**Authors:** Tyler Muffly, T. Chad McCormick, Christopher Cook, Jeffrey Wall

**Affiliations:** Department of Obstetrics and Gynecology, University of Missouri at Kansas City, Kansas City, MO 64108, USA

## Abstract

*Background*. The goal of this case is to review the zoonotic infection, human granulocytic ehrlichiosis, presenting with pyrexia. *Case*. A 22-year-old multigravid female presented to the emergency department with a painful skin rash, high fever, and severe myalgias. The patient underwent a diagnostic evaluation for zoonotic infections due to her geographical and seasonal risk factors. Treatment of human granulocytic ehrlichiosis was successful though the patient spontaneously aborted presumably due to the severity of the acute illness. *Conclusion*. Treatment of human granulocytic ehrlichiosis in pregnancy presents unique challenges. Management of pyrexia during pregnancy is limited to external cooling in the setting of thrombocytopenia and elevated aminotransferases. Extensive counseling regarding teratogenic potential of medications allows the patient to weigh the pros and cons of treatment.

## 1. INTRODUCTION

The tick-borne illness, human granulocytic ehrlichiosis (HGE), is rare during pregnancy. A MEDLINE search from January 1966 through November 2007 using the key words “ehrlichiosis” and “pregnancy” found 21 English language articles. We comment on the therapeutic dilemmas of this unusual combination of hyperthermia and pregnancy that has not been previously described.

## 2. CASE

A 22-year-old multigravid female at seven weeks gestational age presented to the emergency department at a rural referring hospital with symptoms of a rash for three days, vomiting, nonproductive cough, and complaining of “sore throat and burning skin.” The severe burning pain was constant and localized to the areas of the rash. Her prenatal care started at five weeks and was complicated by tobacco use and one episode of suspected streptococcal pharyngitis treated with penicillin six days prior. After evaluation at the referring emergency department, she was transferred to our facility via air medical service.

On examination, the patient was found to be diaphoretic, febrile to 102.1 degrees Fahrenheit, and normotensive. Physical examination showed a diffuse maculopapular rash sparing the palms and soles. A bedside abdominal ultrasound study confirmed fetal movement and a fetal heart rate of 160 beats per minute. Of note, she was leukopenic, thrombocytopenic, and had elevated plasma aminotransferases to ten times normal values. Our suspicion was high for human granulocytic ehrlichiosis due to her presentation from rural Missouri, symptomatology, and classic laboratory findings of leukopenia, thrombocytopenia, and elevated aminotransferases. Thus, with her informed consent, empiric doxycycline treatment was administered. Over the next 24 hours, the patient became progressively more febrile spiking temperatures to 103.9 degrees Fahrenheit.

Over hospital days two and three, her aminotransferases decreased, however she continued to have fevers and the patient remained ill-appearing. The patient’s symptoms of burning skin pain, myalgias, and malaise decreased slowly. On the third day of the hospitalization, she began having vaginal bleeding and passed tissue consistent with a seven-week gestation. Clinical examination and falling beta-HCG levels confirmed a complete abortion. The painful skin rash improved but limited her range of movement. After nine days of doxycycline the patient improved and was discharged to home in good condition. The rash cleared and the patient had no residual symptoms of disease.

## 3. COMMENT

Human granulocytic ehrlichiosis is an underreported tick-borne illness. Ehrlichiosis should be included in the differential diagnosis of any patient presenting with thrombocytopenia, elevation of aminotransferases, painful skin rash, and high fevers. Our patient had no recollection of a tick or insect bite, but she lives in rural Missouri which is mostly wooded containing multiple forms of wildlife. Human granulocytic ehrlichiosis is endemic to Missouri with 0.01–11.9 cases per million persons yearly. Other disease processes with similar pyrogenic presentations include viral exanthems, Epstein-Barr virus, leptospirosis, Rocky Mountain spotted fever, cytomegalovirus, Lyme disease, and Q fever [1]. These illnesses that cause hyperthermia during pregnancy are of particular concern because immunosuppression may cause a more severe disease presentation.

Zoonotic infections of pregnant women have been documented in the literature. Pregnant women outdoors during the spring and fall tend to wear less clothing which may increase their risk for zoonosis. Fever and rash in a pregnant patient are symptoms that should initiate a differential diagnosis including zoonotic infections. Rocky Mountain spotted fever presents in a specific geographic area with a fall and spring temporal distribution. The maculopapular rash in Rocky Mountain spotted fever is similar to that of other tick-borne illnesses. The main clinical factor that discriminates Rocky Mountain spotted fever from HGE is the lack of temperature above 102 degrees Fahrenheit. Leptospirosis has also been reported in pregnant women and is the most common zoonosis worldwide [2]. Conjunctival suffusion is pathognomonic for leptospirosis in a patient with nonspecific febrile illness and myalgias. Exposure to lake, river, and stream water contaminated with animal waste increases the risk of acquiring *Leptospira interrogans* [3]. Q fever is caused by inhalation of infected particles of animal feces. The disease can present with fever, rash, and flu-like illness. Also, Q fever presentation tends to be age-specific, with younger patients developing hepatitis and the older population acquiring pneumonia. *Coxiella burnetti* infection should be considered in patients who have first trimester obstetric complications and fever [4]. Lyme disease manifests with intermittent fevers and chills. The classic Lyme disease rash is an asymptomatic erythema migrans which presents as central clearing within an erythematous base. The list of potential parasitic zoonoses is quite large, however careful history and physical examination can lead to early detection [2].

In the pregnant patient, maternal hyperthermia greater than 103 degrees Fahrenheit denatures proteins causing cell death, membrane disruption, vascular disruption, and placental infarction during organogenesis. Treatment of infectious febrile illnesses during pregnancy can be accomplished with pharmacologic and external cooling methods [5]. However, nonsteroidal anti-inflammatory agents, and acetaminophen are relatively contraindicated antipyretics in a patient with thrombocytopenia and elevated aminotransferases. Antipyretic agents work by lowering the hypothalamic setpoint, which is increased during pyrexia [6]. Endogenous pyrogens, such as interleukin-1 and interleukin-6, cause febrile response by stimulating cerebral prostaglandin-E synthesis [7]. Antipyretic agents block this process by inhibiting the arachidonic acid cycle in the brain [8]. The result is a lowering of the hypothalamic setpoint, which activates the body’s two principle mechanisms for heat dissipation: vasodilation and sweating. External cooling methods attempt to maximize the amount of heat diffused by convection, conduction, and radiation. External cooling methods used to maintain normothermia include water-flow blankets, ice packs to the axillae and groin, intravenous and oral hydration with cool fluids, or immersion in a tepid bath. Shivering causes increased heat production and is a sign to halt cooling efforts.

Some diseases have a limited array of pharmacologic treatments and may potentially cause harm to the fetus. The view that doxycycline, to treat HGE, is relatively contraindicated in pregnancy due to its effects on fetal teeth and bones is no longer accepted as a valid point of medical dogma. Doxycycline is no longer absolutely contraindicated in pregnancy [9]. Alternative treatment for ehrlichiosis during pregnancy includes rifampin in a limited number of patients. Microscopy of buffy coat smear, PCR, and cell culture are ideal methods to confirm the diagnosis of HGE [10]. Wright’s stained buffy coat of peripheral blood will show morulae characteristics on histopathology. Elevated serum antibody titers to HGE can also eliminate rickettsial disease. We suspect that there is much more to be known about the effects of ehrlichiosis on pregnancy.

The case here, with abortion, hyperpyrexia, consumptive coagulopathy, and hepatic involvement, contradicts the recent observation that ehrlichosis was not particularly fulminant in pregnancy. The literature describes human granulocytic ehrlichiosis as a mild illness because of the immunosuppression during pregnancy [11]. The severity of the laboratory findings, clinical presentation, and gestational age make this case unique.
